# VUS-type alteration in POLD1 and microsatellite instability in a metastatic luminal B breast cancer patient

**DOI:** 10.3332/ecancer.2020.1002

**Published:** 2020-01-21

**Authors:** Catarina Marchon da Silva, Denis Shimba, Denise Oishi, Allyne Cagnacci, Ana Carolina Ribeiro Chaves de Gouvea, Felipe Ades

**Affiliations:** 1 Faculdade de Medicina, Universidade Municipal de São Caetano do Sul, São Caetano do Sul, SP 09521-160, Brazil; 2 Centro Especializado em Oncologia, Hospital Alemão Oswaldo Cruz, São Paulo, SP 01323-020, Brazil

**Keywords:** POLD1, luminal B breast cancer, pembrolizumab, microsatellite instability

## Abstract

Microsatellite instability (MSI) and POLD1 mutations are usually described in colorectal tumours in patients with polyposis syndrome but rarely found in breast tumours. This case describes a metastatic luminal B breast tumour in a young patient with an important family history of cancer. Mutational studies found a Variant of Uncertain Significance (VUS)-type alteration in POLD1 that motivated the study for MSI, which was found positive. Recent data point towards the use of pembrolizumab as a treatment option for tumour presenting with MSI instead of chemotherapy.

## Introduction

Breast cancer is a heterogeneous disease with high prevalence in developed and developing countries. Breast cancer has its highest incidence in women between 40 and 70 years old, affecting around 2 million women in 2018 [1]. The disease is divided into four main immunohistochemical profiles, according to receptor expression for oestrogen (ER), progesterone (PgR) and human epidermal 2 growth factor gene receptor (HER2): luminal A, luminal B, Triple-negative and HER2 positive [2]. As a result, treatment is more individualised depending on the subtype.

Microsatellites are nucleotide repetitions of the DNA, responsible for genomic maintenance. During replication, they may suffer errors which are quickly repaired, and normal replication follows. Hence, microsatellite instability (MSI) is the name given to the germline allele portion of the microsatellite that has suffered addition or deletion of its units, product of loss of cell capacity to correct errors associated with replication, resulting in a somatic length alteration [3, 11].

Tumours presenting MSI have higher mutation rates since DNA-repairing genes are either mutated or inactive, resulting in constant DNA exposure to transformation during cell replication. This process leads to neoantigen synthesis, allowing easier recognition by the immune system. Since immunotherapy aims to amplify the immune system while down-regulating tumour-immune evasion mechanisms, it is a useful tool in tumours with these characteristics.

In this scenario, pembrolizumab, an anti-PD-1 monoclonal antibody, prevents lymphocyte down-regulation after tumour PD-L1 and lymphocyte PD-1 binding [4]. As a result, tumour proteins are better recognised for increasing the immune system efficacy and causing cytotoxic cell death. A recent study demonstrated that tumours presenting MSI, regardless of tumour etiology, can be treated with pembrolizumab.

POLD1 is a gene that codes the delta variation of the DNA polymerase, responsible for DNA-associated repair through base excision during cell replication. Its mutation is more typically described in colorectal tumours with MSI, usually in familiar polyposis syndrome patients [5].

As discussed earlier, MSI usually presents itself alongside deficient, mutated or inactive DNA-repair genes; as POLD1 genes code q-repairing enzymes, mutations in this particular gene could be found in MSI tumours. This correlation could explain high rates of tumour mutation and pembrolizumab response rates during treatment regime, as biological and scientific rationale infers that the number of neoantigens in a tumour with deficient q-repair genes and MSI profile would allow amplified immune response—due to immunotherapy—to interfere greatly with cellular proliferation and macroscopically, disease progression.

## Case report

A 42-year-old female patient, G. C. C, no comorbidities, reporting important breast cancer family history, with four first-degree relatives diagnosed with breast cancer under 40 years old. She was diagnosed with breast malignant neoplasm during her pregnancy, undergoing adenomastectomy and sentinel lymph node dissection. The anatomopathological study revealed a grade 3 ductal invasive carcinoma, measuring 0.8 cm with a negative sentinel lymph node. Immunohistochemical profile was ER 85%, PgR (-), HER2 (-), Ki-67 70%, characterising a luminal B tumour (Table 1). At the time of the diagnosis, the patient was advised to undergo genetic testing for BRCA1 and BRCA2 genes, which did not reveal any mutations. She decided not to take adjuvant tamoxifen, radiotherapy nor chemotherapy since she was pregnant at the time of the diagnosis.

**Figure 1.**
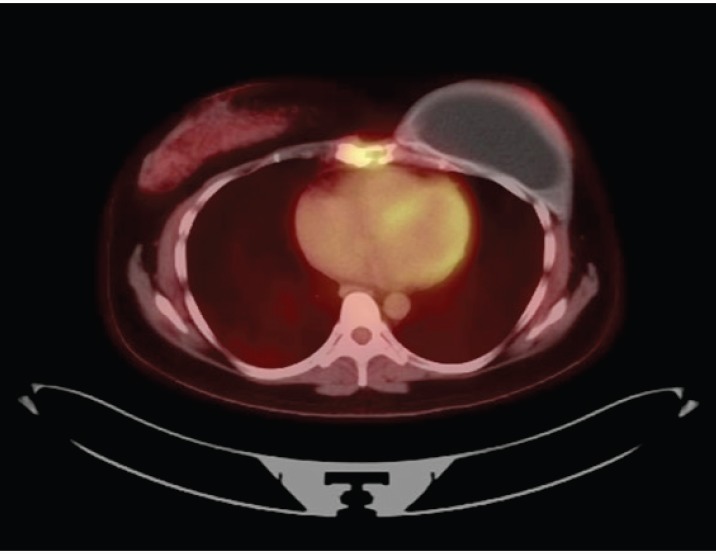
Patient’s PET Scan from 21 November 2019. Axial plane, revealing disease in sternum body.

**Figure 2.**
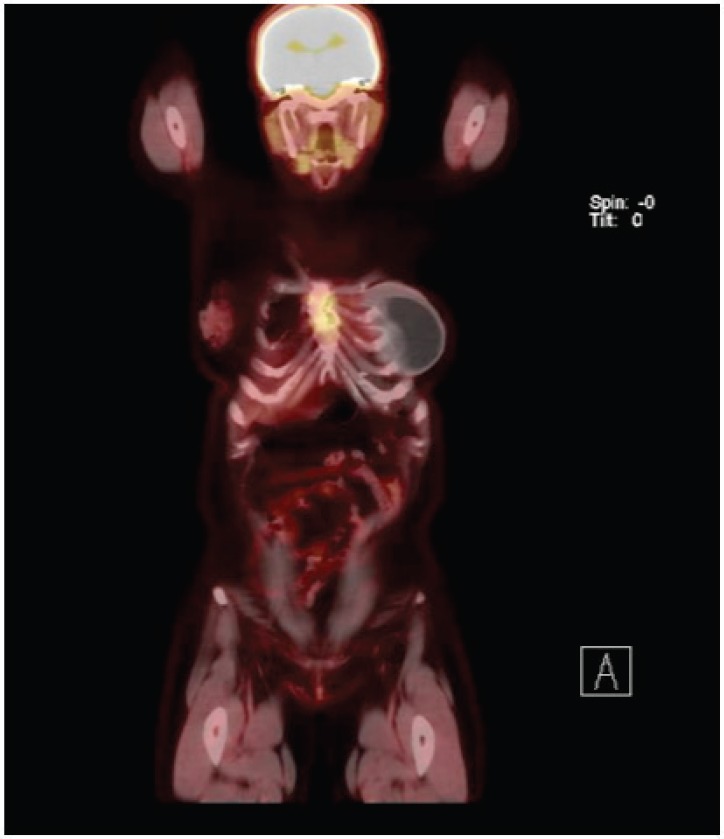
Patient’s PET Scan from 21 November 2019. Sagittal plane, revealing disease in sternum body.

After breastfeeding, she started her treatment with tamoxifen 20 mg/day for 2 years, when she presented disease progression in the sternum body. She was then treated with goserelin, zoledronic acid and exemestane. In addition, she received radiotherapy in the sternum region, as this was her only metastatic lesion. She had stable disease for 3 years when a PET-CT scan showed increased FDG-uptake in the same region of the sternum body (Figures 1 and 2). She was treated locally with cryotherapy and continued with the same systemic regimen. After another 2 years of disease control, a new PET-CT scan shown mixed-pattern lesions on the upper half of the sternum body with bone cortical erosion and small soft-tissue component in the anterior and lateral margins, measuring 5 cm with standarized uptake value (SUV) = 4.4. Physical examination exhibited a painful lump in the sternum bone, compatible with the PET/CT image. Treatment was then changed to intramuscular fulvestrant 500 mg + leuprorelin every 28 days, with complete shrinkage of the sternum lump and symptom improvement. The patient has now stable disease for almost 2 years since starting this treatment line.

Due to important family history and young age at the time of diagnosis, a full germline mutation panel was ordered to investigate further mutations associated. The panel detected for a VUS-type alteration in POLD1 gene – c.1923dupC (p.Thr642Hisfs*97) – kind of mutation is possibly associated with MSI. As high expression of POLD1 is associated with poor prognosis [6], and there are therapeutic implications to this, tumoural fragments from the primary tumour were then tested by immunohistochemistry for mismatch repair (MMR) deficiencies, which revealed a MSH6 instability, characterising MSI phenotype (Table 2). These recent findings may explain the indolent curse of the disease and open the possibility of treatment with immunotherapy in subsequent lines of treatment.

## Discussion

This is a luminal B breast cancer case with a typical presentation at diagnosis in which mutation testing broadened the possibilities of treatment options. POLD1 germline mutations are rare in breast cancer, as it is more commonly found in colorectal cancer patients with polyposis syndrome [5].

Interestingly, POLD1 mutations are described interacting with BRCA1 and BRCA2 genes [7], increasing the risk for allelic mutations, leading to increased chances of tumour development, which was not seen in the previously described case. Furthermore, the presence of MSI, associated with POLD1 alterations or without is rarely described in breast malignant tumours [8], and in the case of luminal tumours it was previously described with lower overall survival and worst prognosis [9].

Tumours with MSI usually present a high tumoural mutational burden, often associated with the presence of neoantigens and higher response to treatment with immunotherapy. The use of pembrolizumab was tested in a phase II study including 41 patients with colon cancer with or without MSI and non-colorectal cancer with MSI. The response rate and progression-free survival (PFS) rate was 40% and 78% in the deficient cohort and 0% and 11% in the proficient cohort. The median PFS and median overall survival were not reached in the deficient cohort and 2.2 and 5.0 months in the proficient cohort. Whole-exome sequencing revealed a mean 1,782 somatic mutations per tumour in the MSI cohort and 73 mutation in the proficient cohort. High somatic mutations were associated with longer PFS [9].

This is a rare case of VUS-type alteration in POLD1 and MSI in breast cancer. The discovery of the VUS-type alteration partnered with the investigation for MSI opens new treatment opportunities for this patient. In the future, molecular testing will allow better understanding of disease biology and tailor therapy for each specific individual.

The complexity of interactions between somatic and germline mutations in the carcinogenic behaviour of cancer and disease heterogeneity poses a challenge to the future investigation of cancer treatment. However, with the accumulating real life and study data added to translational and preclinical studies on cancer heterogeneity, we are starting to unveil some of the mechanism and pave the way towards more personalised medicine.

## Conclusion

Finally, the use of pembrolizumab on solid tumours with MSI leads to better tumour response and control if not complete response [4] and could be considered as an option for patients who are of young age and with tumours displaying such characteristics. The medication may be able to stop disease progression while maintaining quality of life and presenting fewer side effects [11].

Since there are few described cases of POLD1 mutation and MSI in breast cancer, pembrolizumab may be one more treatment option for the reported patient, reserving the use of chemotherapy for subsequent treatment lines, if needed. Despite these findings and new possibilities, more studies focusing on the relationship between the use of pembrolizumab in tumours with POLD1 mutation and MSI in breast cancer, in general, are required.

## Conflicts of interest

The authors have declared no relevant conflicts of interest related to this work.

## Funding declaration

The authors have received no funds for the development of this work.

## Figures and Tables

**Table 1.** Tumour immunohistochemistry illustrating a luminal B tumour phenotype.

Left Breast		
**Antibody**	**Clone**	**Interpretation**
EGFR	31G7	Negative in the tumor cells
Progesterone Receptor (PR)	PgR636	Negative in the tumor cells
Estrogen Receptor (ER)	SP1	Positive in 85% of the tumor cells
c-erB-2	SP3	Escore 0
Ki-67	MIB-1	Positive in 70% of the tumor cells
CK5	Polyclonal	Negative in the tumor cells

**Antigen**	**Result**	
Estrogen Receptor	Positive (moderate intensity, 70% of the cells)	
Progesterone Receptor	Negative	
HER-2	Negative	
Ki-67 (MIB-1)	Positive (30%)	
Cytokeratin 5	Negative	
TTF-1	Negative	
Cytokeratin 7	Positive	
Cytokeratin 20	Negative	
Mammaglobin	Negative	

**Table 2.** Immunohistochemistry revealing MMR instability in MSH6, characterizing MSI phenotype. Result issued on 27 March 2019.

Antibodies	
MLH1	Nuclear reaction preserved = ‘stable’ profile
MSH2	Nuclear reaction preserved = ‘stable’ profile
MSH6	Loss of reaction = ‘unstable’ profile
PMS2	Nuclear reaction preserved = ‘stable’ profile

## References

[1] International Agency for Research on Cancer, World Health Organization (2019). Estimated number of new cases in 2018, worldwide, both sexes, all ages. GLOBOCAN.

[2] Sarturi PR, Ademar DC, de Morais CF (2012). Perfil imunohistoquímicodo câncer de mama de pacientes atendidas no Hospital do Câncer de Cascavel-Paraná. Rev Bras Onco Clínica.

[3] Lopez G, Corti C, Pesenti C (2018). Mismatch repair protein loss as a prognostic and predictive biomarker in breast cancers regardless of microsatellite instability. JNCI Cancer Spectrum.

[4] Le DT, Uram JN, Wang H (2015). PD-1 blockade in tumors with mismatch-repair deficiency. N Engl J Med.

[5] Sigurdson AJ, Hauptmann M, Chatterjee N (2004). Kin-cohort estimates for familial breast cancer risk in relation to variants in DNA base excision repair, BRCA1 interacting and growth factor genes. BMC Cancer.

[6] Qin Q, Tan Q, Li J (2018). Elevated expression of POLD1 is associated with poor prognosis in breast cancer. Oncol Lett.

[7] Valle L, Hernández-Illán E, Bellido F (2014). New insights into POLE and POLD1 germline mutations in familial colorectal cancer and polyposis. Hum Mol Genet.

[8] Anbazhagan R, Fujii H, Gabrielson E (1999). Microsatellite instability is uncommon in breast cancer. Clin Cancer Res.

[9] de la Chapelle A (2003). Microsatellite Instability. N Engl J Med.

[10] Wang F, Zhao Q, Wang Y-N (2019). Evaluation of POLE and POLD1 mutations as biomarkers for immunotherapy outcomes across multiple cancer types. JAMA Oncol.

[11] University of Pittsburgh Schools of the Health Sciences (2016). Immunotherapy improves survival, quality of life in rapidly progressing head and neck cancer. ScienceDaily.

[12] Jansen AM, van Wezel T, van den Akker BE (2016). Combined mismatch repair and POLE/POLD1 defects explain unresolved suspected Lynch syndrome cancers. Eur J Hum Genet.

